# Geometric isomers of di­chlorido­iron(III) com­plexes of CTMC (5,7,12,14-tetra­methyl-1,4,8,11-tetra­aza­cyclo­tetra­deca­ne)

**DOI:** 10.1107/S205322962200849X

**Published:** 2022-08-30

**Authors:** Stephanie S. DeLancey, Reese A. Clendening, Matthias Zeller, Tong Ren

**Affiliations:** a Department of Chemistry, Purdue University, 560 Oval Dr., W. Lafayette, IN 47907-2084, USA; Sun Yat-Sen University, People’s Republic of China

**Keywords:** stereoisomerism, iron(III) macrocyclic com­plexes, geometric com­plex control, crystal structure, tetraazacyclotetradecane, CTMC

## Abstract

Both *trans* and *cis* iron–CTMC com­plexes, namely, *trans*-di­chlorido­[(5*SR*,7*RS*,12*RS*,14*SR*)-5,7,12,14-tetra­methyl-1,4,8,11-tetra­aza­cyclo­tetra­deca­ne]iron(III) tetra­chlorido­ferrate, [Fe(C_14_H_32_N_4_)Cl_2_][FeCl_4_] (**1a**), the analogous chloride methanol monosolvate, [Fe(C_14_H_32_N_4_)Cl_2_]Cl·CH_3_OH (**1b**), and *cis*-di­chlo­rido­[(5*SR*,7*RS*,12*SR*,14*RS*)-5,7,12,14-tetra­methyl-1,4,8,11-tetra­aza­cyclo­tetra­dec­ane]iron(III) chloride, [Fe(C_14_H_32_N_4_)Cl_2_]Cl (**2**), were successfully synthesized and structurally characterized using X-ray diffraction. The coordination geometry of the macrocycle is dependent on the stereoisomerism of CTMC. The packing of these com­plexes appears to be strongly influenced by extensive hydro­gen-bonding inter­actions, which are in turn determined by the nature of the counter-anions (**1a** *versus* **1b**) and/or the coordination geometry of the macrocycle (**1a/1b** *versus* **2**). These observations are extended to related ferric *cis*- and *trans-*di­chloro macrocyclic com­plexes.

## Abstract 2

Di­chlorido­iron(III) com­plexes of two stereoisomers of a tetra­aza­macrocycle are examined. The stereoisomerism of the macrocycle is shown to determine the geometric isomerism of the resulting metal com­plex, and these results are com­pared to relevant previous reports.

## Introduction

Macrocyclic com­plexes of transition metals, due to the inherent stability imparted by the macrocyclic effect (Constable, 1999 ▸), as well as to the ability to tune the number and position of open coordination sites, have often served as model com­plexes for the study of a number of chemical phenomena. For example, simple tetra­aza­macrocycles containing iron have been used to investigate aspects of nitro­gen fixation (Meyer *et al.*, 1999 ▸), nonheme oxoiron (Prakash *et al.*, 2015 ▸; Rohde *et al.*, 2003 ▸), CO_2_ reduction (Straub & Vöhringer, 2021 ▸), and water oxidation catalysis (Kottrup & Hetterscheid, 2016 ▸). Importantly, the macrocycle may often adopt either a folded or planar coordination geometry, leaving either *cis*- or *trans*-open coordination sites, respectively. This geometry can dramatically influence the reactivity of the resulting com­plexes (Kottrup & Hetterscheid, 2016 ▸; Meyer *et al.*, 1999 ▸).

Our group has studied iron–alkynyl com­plexes supported by cyclam (1,4,8,11-tetra­aza­cyclo­tetra­deca­ne) (Cao *et al.*, 2012 ▸), HMC (5,5,7,12,12,14-hexa­methyl-1,4,8,11-tetra­aza­cy­clo­tetra­deca­ne) (Clendening *et al.*, 2022 ▸), and an HMC-derived tetra­imine com­plex (HMTI = 5,5,7,12,12,14-hexa­methyl-1,4,8,11-tetra­aza­cyclo­tetra­deca-1,3,8,10-tetra­ene) (Clen­dening & Ren, 2022 ▸) as models for potential mol­ecular wires. The macrocycle has been shown to strongly affect the properties of these com­plexes, even tuning the metal–alkynyl bonding. Although Cr^III^–alkynyl com­plexes with the macrocycle in both folded and planar conformations have been characterized (Tyler *et al.*, 2016 ▸), iron–alkynyl com­plexes were only isolated with planar macrocycles, even when starting from a *cis*-Fe^III^(cyclam) com­plex. Seeking to expand our library of com­pounds, we have recently turned to the investigation of Fe(CTMC) com­plexes (CTMC = 5,7,12,14-tetra­methyl-1,4,8,11-tetra­aza­cyclo­tetra­deca­ne). Nickel com­plexes of CTMC (and other macrocycles; Wang *et al.*, 2019 ▸) have received considerable attention, most recently as efficient carbon dioxide reduction catalysts (Mash *et al.*, 2019 ▸), but no iron com­plexes of CTMC have been reported previously to our knowledge. 
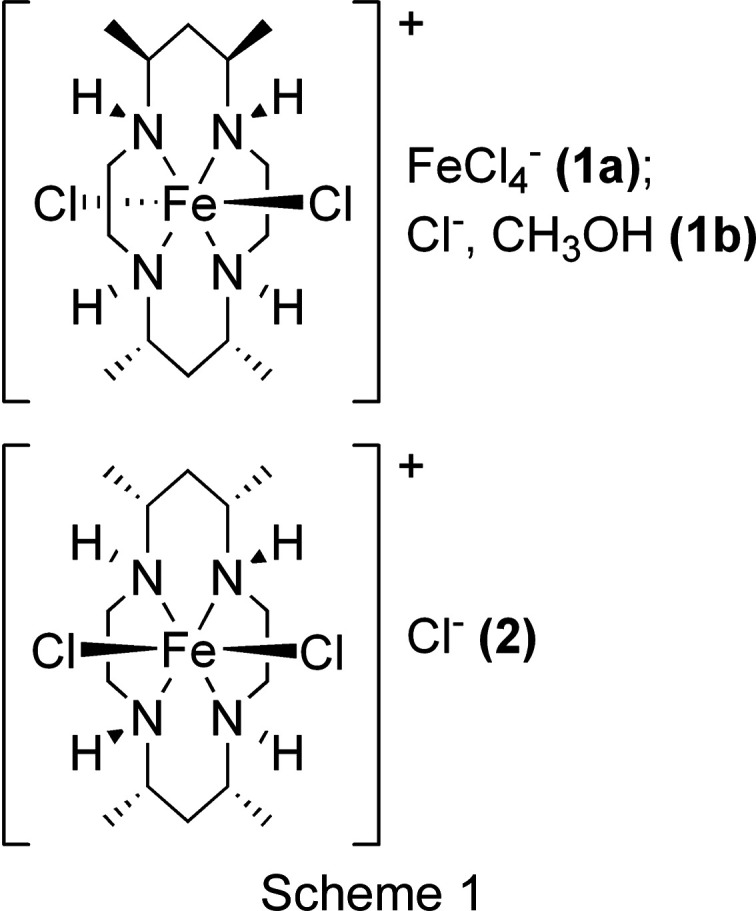


**Figure 1:**
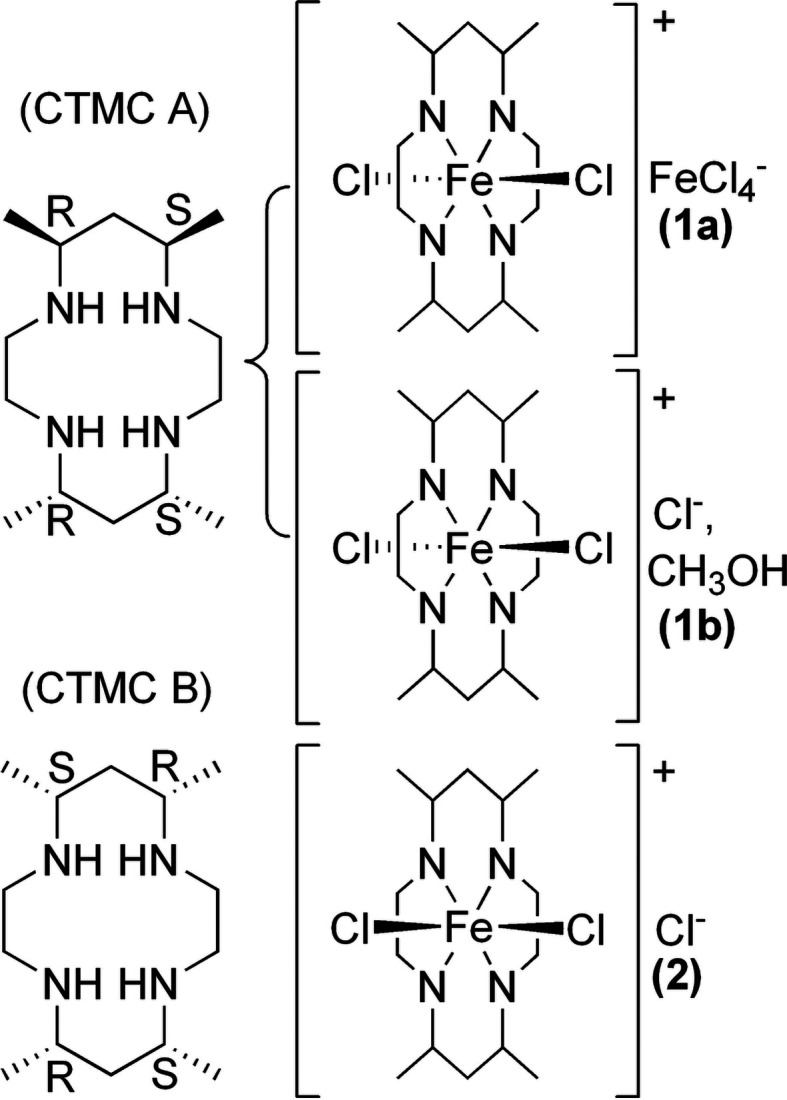
Stereoisomers of CTMC (left) and the resulting iron coordination com­plexes (right) discussed herein.

Although CTMC is known to form as a mixture of stereoisomers, the vast majority of structures contain only isomer **A** [(5*SR*,7*RS*,12*SR*,14*RS*)-5,7,12,14-tetra­methyl-1,4,8,11-tetra­aza­cyclo­tetra­decane; Fig. 1 ▸]. The structure of the free ligand has been determined, which contained **A** and **B** [Fig. 1 ▸; **B** = (5*SR*,7*RS*,12*SR*,14*RS*)-5,7,12,14-tetra­methyl-1,4,8,11-tetra­aza­cyclo­tetra­deca­ne] (Tahirov *et al.*, 1995*a* ▸). Finally, the structure of an Ni^II^(CTMC) com­plex has been reported containing a third stereoisomer, **C** [(5*SR*,7*RS*,12*RS*,14*RS*)-5,7,12,14-tetra­methyl-1,4,8,11-tetra­aza­cyclo­tetra­deca­ne] (Tahirov *et al.*, 1995*b* ▸). Notably, all the structures of metal-ion com­plexes of CTMC reported to date contain a planar macrocycle.

Here we report the first examples of iron–CTMC com­plexes and their crystallographically determined structures, namely, *trans*-di­chlorido­[(5*SR*,7*RS*,12*RS*,14*SR*)-5,7,12,14-tetra­methyl-1,4,8,11-tetra­aza­cyclo­tetra­deca­ne]iron(III) tetra­chlorido­fer­rate (**1a**), the analogous chloride methanol monosolvate (**1b**), and *cis*-di­chlorido­[(5*SR*,7*RS*,12*SR*,14*RS*)-5,7,12,14-tetra­methyl-1,4,8,11-tetra­aza­cyclo­tetra­deca­ne)iron(III) chloride (**2**). While **1a** and **1b** exhibit the common stereoisomer **A** with a planar macrocyclic conformation, **2** represents the first structurally characterized metal CTMC com­plex both with stereoisomer **B** and with a folded macrocycle conformation (Fig. 1 ▸). Thus, the stereoisomer of the macrocycle appears to control the coordination isomer of the resulting metal com­plex. Given the importance of macrocycle coordination geometry on the properties of the resultant com­plex, and the extensive literature on CTMC species, we describe here the structural variations among these species, specifically addressing the differences between the folded (*cis*-di­chloro com­plex) and planar (*trans*-di­chloro com­plexes) coordination motifs. As will be established below, the number and stereochemistry of the macro­cyclic substituents can dramatically alter the iron–macro­cycle bonding inter­actions. This work thus serves as a potential entry point for the development of novel Fe analogues of known com­plexes while exercising fine-tuned steric and electronic control over the Fe–macrocyclic core.

## Experimental

The CTMC macrocycle was synthesized as described in the literature (Kolinski & Korybut-Daszkiewicz, 1975 ▸; Mash *et al.*, 2019 ▸; Tahirov *et al.*, 1995*a* ▸). Consistent with previous structure determinations of the free ligand (Tahirov *et al.*, 1995*a* ▸), we found evidence for the presence of at least two stereoisomers of CTMC (**A** is 5*SR*,7*RS*,12*SR*,14*RS* and **B** is 5*SR*,7*RS*,12*SR*,14*RS*), confirmed by the structures determined for the re­sulting Fe^III^ com­plexes reported herein. Two batches of CTMC were used to generate the *trans* and *cis* iron CTMC chloride species, with **A** being dominant in batch one (m.p. 178–181 °C) and **B** dominant in batch two (m.p. 70–135 °C). Altering the experimental method did not seem to allow for control over the presence of either stereoisomer of CTMC, and attempts to separate **A** and **B** were unfruitful. This lack of selectivity is consistent with the reported cocrystallization of **A** and **B** from the same synthesis (Tahirov *et al.*, 1995*a* ▸). All commercially available materials were used as received. The IR spectra (ATR) of **1a**, **1b**, and **2** as powders were collected on a JASCO FT–IR 6300 instrument equipped with a diamond crystal. Magnetic measurements were conducted using a Johnson Matthey Magnetic Susceptibility Balance. Electron-spray ionization mass spectrometry experiments were performed with an Advion Mass Spectrometer.

### Synthesis and crystallization

#### Synthesis of *trans*-[Fe(CTMC)Cl_2_][FeCl_4_]/Cl (1a/1b)

CTMC (0.449 g, 1.75 mmol, batch one) was dissolved in a 2:1 (*v*/*v*) di­methyl­formamide–triethyl orthoformate mixture (30 ml) prior to purging with nitro­gen. The mixture was stirred and heated while FeCl_2_·4H_2_O (0.697 g, 3.50 mmol) was dis­solved in a 4:3 (*v*/*v*) di­methyl­formamide–triethyl orthoformate mixture (35 ml), previously purged with nitro­gen, and was also left to stir and heat. The CTMC solution was transferred to the iron-containing flask *via* a cannula and the reaction was left to stir at 50 °C for 1 h. The reaction mixture was then exposed to oxygen and about 2 ml of con­centrated hydro­chloric acid were added dropwise to the flask while bubbling through oxygen. An additional 3 ml of con­centrated hydro­chloric acid were subsequently added, along with excess diethyl ether. A yield of 0.333 g of green powder was recovered *via* filtration and crystals of **1a** (space group *C*2/*c*; orange blocks) and **1b** (*P*2_1_/*c*; yellow needles) were grown *via* slow diffusion of diethyl ether into a con­centrated methanol solution of the product. ESI–MS: [*M*]^+^, 381.9 (^35^Cl_2_), 384.0 (^35^Cl, ^37^Cl). IR (cm^−1^): N—H 3174 (*m*), 3116 (*m*). μ_eff_ = 4.6 µ_B_ for the mixture of **1a/1b**, assuming a mol­ecular weight of 418.63 g for [Fe(CTMC)Cl_2_]Cl.

#### Synthesis of *cis*-[Fe(CTMC)_2_]Cl (2)

CTMC (0.199 g, 0.775 mmol, batch two) was dissolved in a 3:1 (*v*/*v*) di­methyl­formamide–triethyl orthoformate solution (16 ml) and then purged with nitro­gen. In a second flask, FeCl_2_·4H_2_O (0.311 g, 1.57 mmol) was dissolved in about 17 ml of a 2:1 (*v*/*v*) di­methyl­formamide–triethyl orthoformate solution and the resulting solution was purged with nitro­gen. Both solutions were warmed and the CTMC solution was transferred *via* cannula to the iron material flask and the reaction mixture was left to stir under nitro­gen at 55 °C for 45 min. Oxygen was then bubbled through the solution while 1 ml of con­centrated hydro­chloric acid was added. The slurry which formed was left to stir at room tem­per­ature for 2 h and 0.126 g of orange powder was obtained after filtration and washing with diethyl ether (30.1% yield). Compound **2** crystallized in the space group *Pbcn* from the slow diffusion of acetone into an aqueous solution of the product. ESI–MS: [*M*]^+^, 382.0 (^35^Cl_2_), 384.0 (^35^Cl,^37^Cl). IR (cm^−1^): N—H 3073 (*m*), 3125 (*sh*). μ_eff_ = 5.6 µ_B_.

### Refinement

Crystal data, data collection and structure refinement details are summarized in Table 1 ▸. H atoms were placed in calculated positions, riding on the parent atom, except for the amine H atoms of **1a** (H1 and H2) and **1b** (H1–H4), which were refined. Methyl and hy­droxy (methanol) groups were permitted to rotate. *U*_iso_(H) values were set to a multiple of *U*_eq_(C,N,O), with 1.5 for CH_3_ and OH, and 1.2 for CH, CH_2_, and NH units, respectively. The Fe atom of the FeCl_4_^−^ counter-anion in **1a** was modeled with very minor disorder over two positions (both located on two­fold rotation axes) with close to identical but slightly shifted positions for the Cl atoms. The anisotropic displacement parameters (ADPs) of the Fe atoms (related by a half unit-cell shift with identical orientations) were constrained to be identical. *U^ij^* com­ponents of ADPs for the Cl atoms were pairwise restrained to be similar (using a SIMU restraint with both s.u. values set to 0.01 Å^2^). The occupancy ratio refined to 0.9803 (7):0.0197 (7). The cationic moiety of **2** exhibits disorder about a *pseudo*-mirror plane through the metal center. Both disordered moieties have crystallographic two­fold symmetry with half the cation within the asymmetric unit. Exempt from the disorder are the Fe atom and the noncoordinated chloride counter-anion (Cl2). The disordered moieties were restrained to have similar anisotropic displacement parameters and 1,2 and 1,3 bond distances and angles. The C1—N1 and C1*B*—N1*B* bond lengths required additional restraints. The occupancy ratio refined to 0.944 (3):0.056 (3). Notably, the minor disordered moiety maintains the hydro­gen-bonding network evident between the major cationic moiety and the chloride counter-anion (*vide infra*), which may facilitate the presence of the minor moiety.

### Computational details

The cationic portions of **1** and **2** were optimized from the crystallographic coordinates of **1a** and **2** (counter-anions were omitted) in the *GAUSSIAN16* suite (Frisch *et al.*, 2016 ▸) using density functional theory (DFT) under vacuum, with the B3LYP functional (Becke, 1993 ▸) and Def2-SVP basis set (Weigend & Ahlrichs, 2005 ▸). The experimental magnetic moment of **1a**/**1b** (*vide supra*) is ambiguous, particularly given the presence of FeCl_4_^−^; therefore, both high- and low-spin states were tested: the low-spin state was found to be lowest in energy for **1** and was thus used for all remaining calculations on structures with a planar macrocycle. It was only possible to optimize the high-spin state for **2** (consistent with the experimental magnetic moment); thus, this was the only spin state considered for structures with a folded macrocycle. *GaussView6* (Dennington *et al.*, 2016 ▸) was used to analyze the data and manipulate the structures to form inter­mediate stereoisomers, as discussed below (see Fig. 6 ▸ for relevant discussion).

## Results and discussion

**Figure 2:**
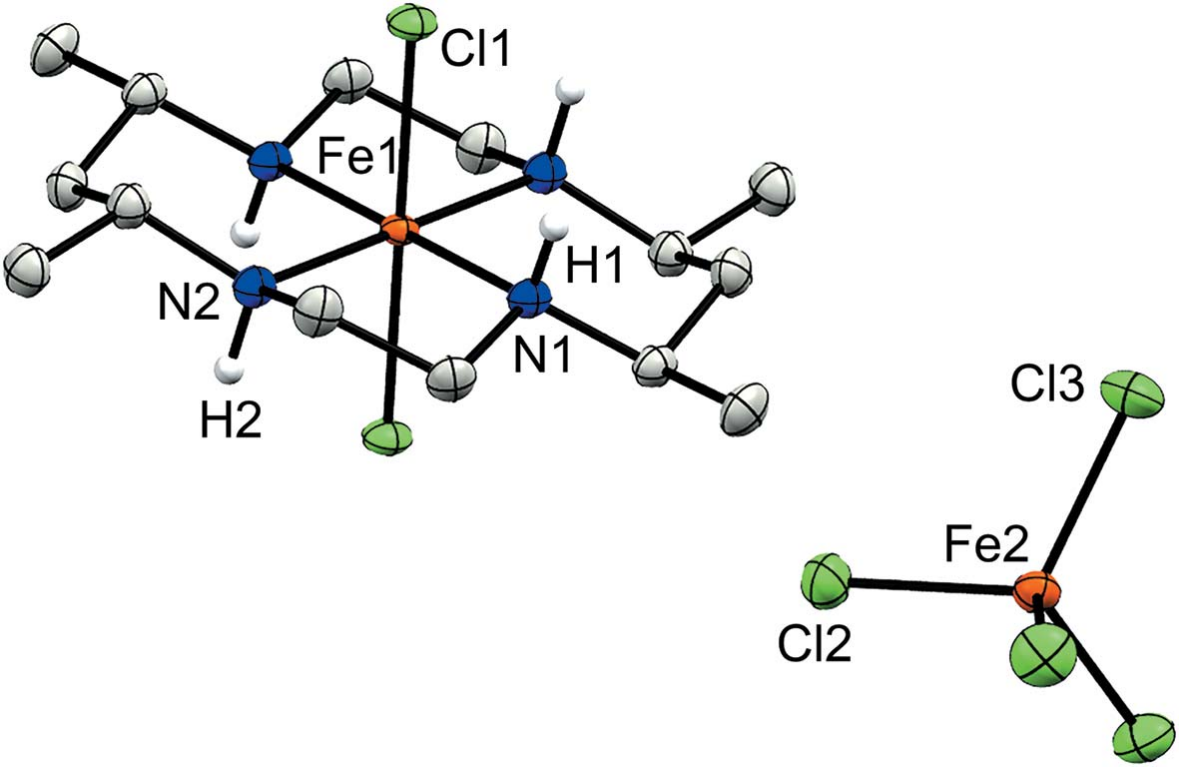
Displacement ellipsoid plot of **1a**. H atoms (except for those bound to N atoms) and the minor disordered FeCl_4_^−^ moiety have been omitted for clarity.

**Figure 3:**
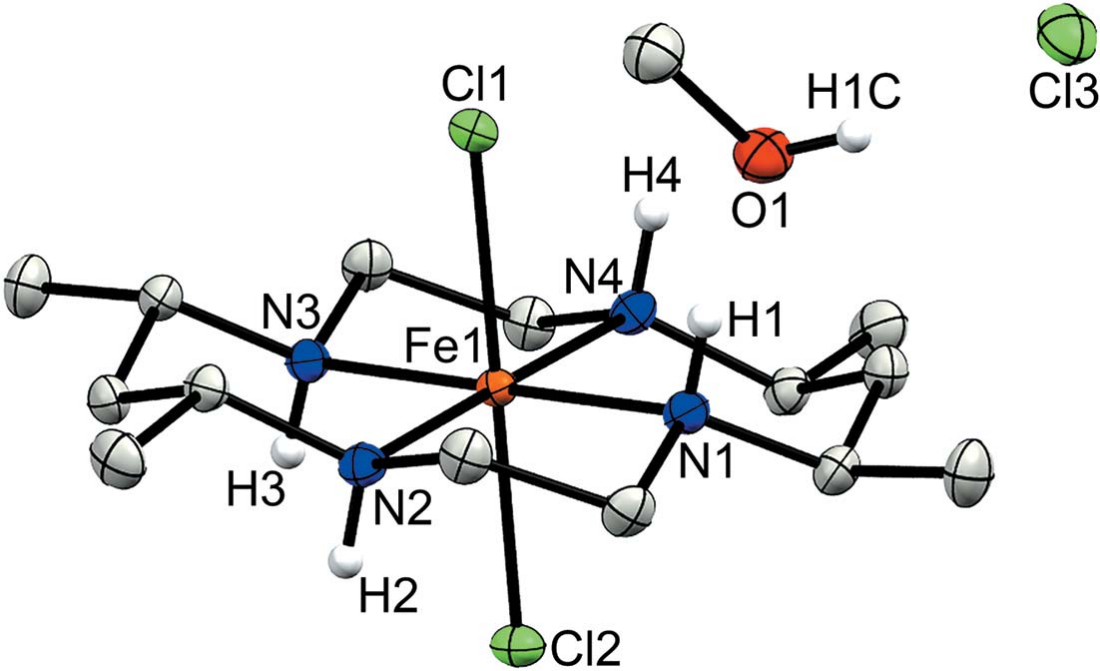
Displacement ellipsoid plot of **1b**. H atoms (except for those bound to N and methano­lic O atoms) have been omitted for clarity.

**Figure 4:**
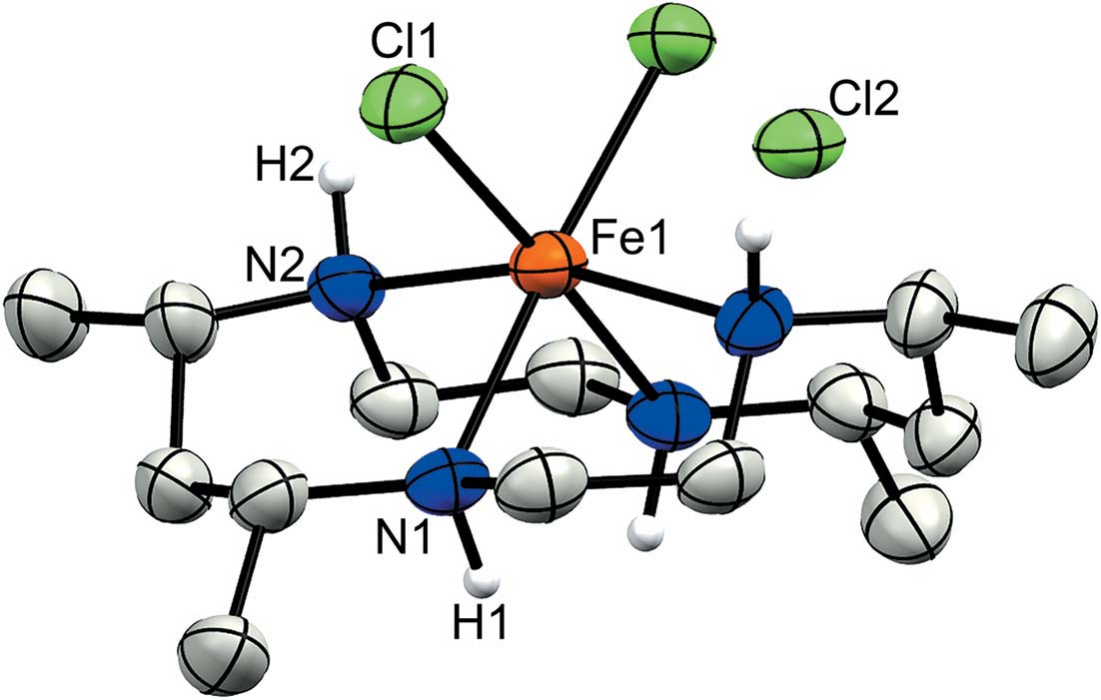
Displacement ellipsoid plot of **2**. H atoms (except for those bound to N atoms) and the minor disordered moiety have been omitted for clarity.

Complex **1a** crystallized in the space group *C*2/*c* with the center of mass of the disordered FeCl_4_^−^ counter-anion lying on a two­fold rotation axis, while the Fe atom of the macrocyclic moiety (Fe1) lies on an inversion center (Fig. 2 ▸). In contrast, no atoms lie on special positions in com­plex **1b**, which crystallized in the space group *P*2_1_/*c* (Fig. 3 ▸) with a methanol solvent mol­ecule. Finally, com­plex **2** crystallized in the space group *Pbcn*, with both the chloride counter-anion and iron center lying on a two­fold rotation axis (Fig. 4 ▸).

**Figure 5:**
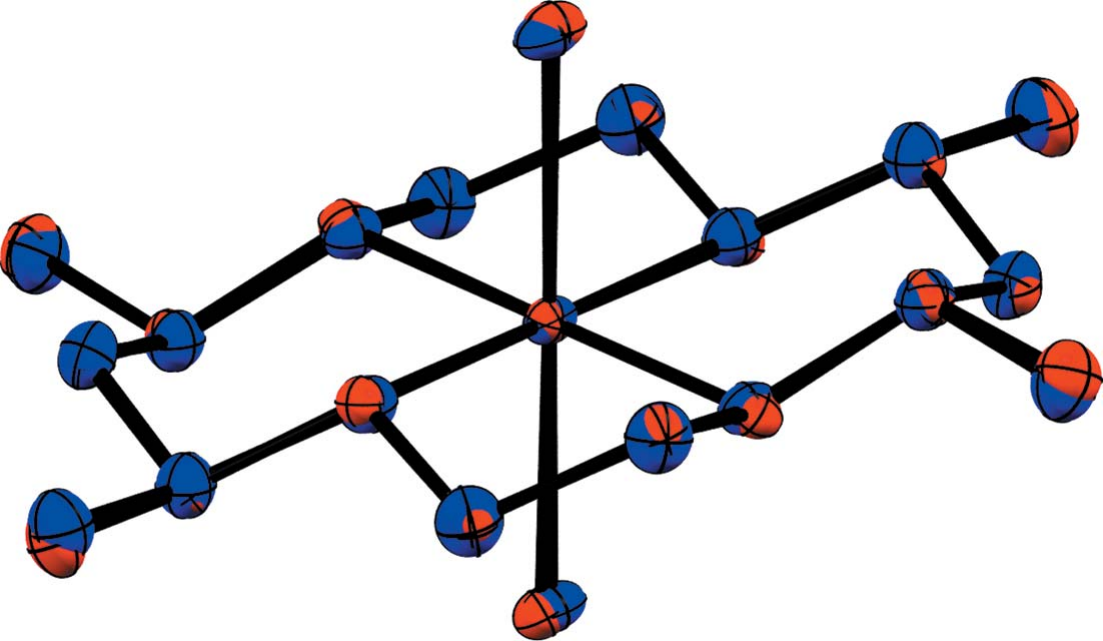
Least-squares overlay of **1a** (blue) and **1b** (red).

The Fe centers in all three com­plexes display a pseudo-octa­hedral geometry with four coordination sites occupied by the N atoms of the CTMC macrocycle and two by the chloride ligands. Additionally, regardless of *cis*- or *trans*-di­chloro config­uration, **1** and **2** both display similar Fe—Cl bond lengths ranging from 2.2710 (3) Å in **1a** to just greater than 2.30 Å in **1b** and **2** (see Table 2 ▸ for selected bond lengths and angles). As expected, each structure exhibits larger N—Fe—N bond angles between amines bridged by the CH(Me)CH_2_CH(Me) linkage and smaller angles between the CH_2_CH_2_ linkage of the macrocycle. Although the structural parameters of the cation in **1b** are very similar to those of **1a**, the Fe—N and Fe—Cl bond lengths are slightly elongated in the former, which may be due to the differences in packing (*vide infra*). The least-squares overlay of **1a** and **1b** (Fig. 5 ▸) illustrates the general similarity of the cationic moieties. Both **1b** and **2** possess a noncoordinated chloride counter-ion, while **1a** contains a tetra­chlorido­ferrate ion. As noted in the *Experimental* (Section 2), **2** was crystallized with the cationic portion disordered over two positions *via* a *pseudo*-mirror plane. As the second moiety accounts for only 5.6 (3)% of the occupancy and its bond lengths and angles were restrained to match that of the major moiety, it is not considered in the general bond length and angle com­parisons.

The CTMC macrocycle appears as stereoisomers **A** and **B** (Fig. 1 ▸), as noted above. It is clear from the structures of **1a**, **1b**, and **2** that **A** tends to yield an iron com­plex in the *trans* configuration (**1a**/**1b**), while **B** tends to yield a com­plex in the *cis* configuration (**2**). To the best of our knowledge, the structure of **2** is the only example of a CTMC com­plex of **B**, and the only structure of a com­plex with a folded CTMC macrocycle. As seen in Table 2 ▸, the Fe—N bond lengths are consistently longer for **2** [2.154 (4)–2.213 (4) Å] com­pared to **1a** [2.0203 (11)–2.0276 (11) Å] and **1b** [2.0654 (8)–2.0826 (9) Å]. The N—Fe—N bond angles in **2** also deviate further from the ideal octa­hedral geometry of 90° than the same bond angles in **1a** and **1b**. Overall, the data suggest that the folded macrocycle conformation in **2** results in some strain com­pared to the planar coordination seen in **1a**/**1b**, favoring weaker Fe—N bonds.

To rationalize the preferences of a given stereoisomer for planar or folded coordination about the metal center, a series of DFT calculations were com­pleted with **1a** as a low-spin com­plex and **2** as a high-spin com­plex in the gas phase. These calculations were com­pleted by rotating one and then two methyl groups on the CTMC macrocycle, effectively reversing the *R* or *S* designation of the methyl groups, until the macro­cycle was converted into the other observed stereoisomer (*i.e.* **A** into **B** and *vice versa*).

**Figure 6:**
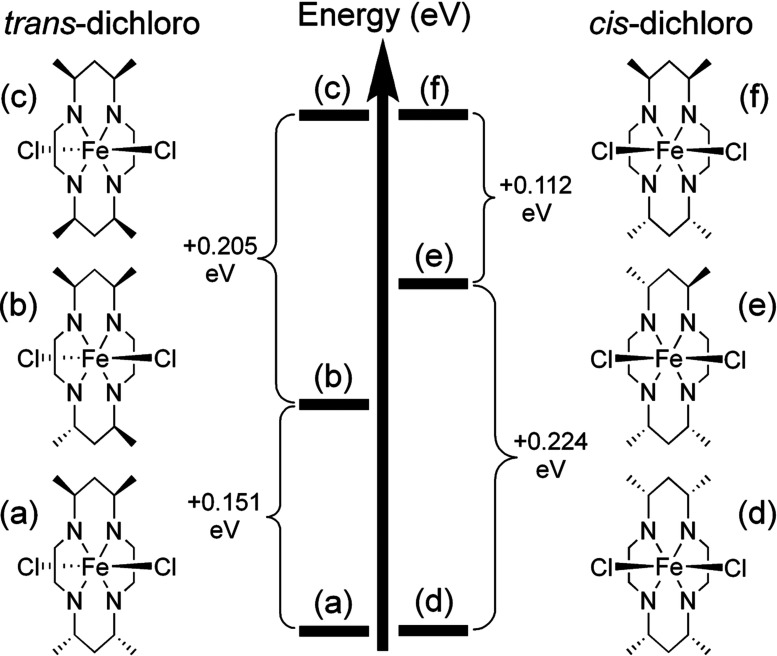
DFT-derived energetic orderings for com­plexes of various isomers of CTMC in planar (left) or folded (right) conformations.

The DFT calculations indicate that the energy of the *cis* and *trans* configurations are indeed related to the stereoisomer of the macrocycle. A *trans* com­plex with **B** [Fig. 6 ▸(*c*)] is calculated to have an energy nearly 0.36 eV greater than that of a *trans* com­plex with **A** [*i.e.* **1a**/**1b**; Fig. 6 ▸(*a*)]. Forcing **A** to assume a folded conformation about the metal center [Fig. 6 ▸(*f*)] results in a calculated energy nearly 0.34 eV higher than that of the folded coordination of stereoisomer **B**. The energies of all *trans*-di­chloro structures [Figs. 6 ▸(*a*)–(*c*)] are calculated to be lower than those of all *cis*-di­chloro structures [Figs. 6 ▸(*d*)–(*f*)]. However, this is likely an artifact due to the different spin states (see *Experimental*, Section 2) assigned for the *trans* (*a*–*c*, *S* = 1/2) and *cis* (*d*–*f*, *S* = 5/2) geometries. Moreover, these gas-phase calculations neglect the counter-anions, with which the cations inter­act substanti­ally in the solid state (*vide infra*). Nevertheless, the stepwise trends within each geometric series (*a*–*c* and *d*–*f*) clearly illustrate the dependence of the energy of the planar and folded coordination modes on the stereoisomer of CTMC. Consistent with **1a**/**1b**, **A** clearly prefers a planar coordination geometry. In contrast, a folded macrocycle is most easily obtained with **B**, consistent with the structure of **2**. These energetic preferences are most reasonably attributed to the effect of the methyl substituents, which lean towards the arrangement of minimal steric inter­actions. This is consistent with reports on metal com­plexes of HMC, for which a strong preference of the stereoisomers to form *cis* or *trans* metal com­plexes is well established (Clendening *et al.*, 2019 ▸; House *et al.*, 1983 ▸; Tyler *et al.*, 2016 ▸).

The previously reported data for *cis*/*trans*-[Fe^III^(cyclam)Cl_2_]^+^ and *cis*/*trans*-[Fe^III^(HMC)Cl_2_]^+^ are useful for the discussion pertaining to planar *versus* folded macrocycles (Clendening *et al.*, 2019 ▸; Guilard *et al.*, 1997 ▸). As with iron–CTMC, the Fe—Cl bond lengths of these Fe^III^ com­plexes remain similar to one another regardless of a *cis* or *trans* nature. These com­plexes display Fe—Cl bond lengths ranging from 2.27 to 2.32 Å, with no significant dependence on the coordination geometry of the macrocycle. In contrast, the Fe—N bond lengths for all of the com­plexes confirm the trends observed for **1a**/**1b** *versus* **2**. Similar to the difference (0.16 Å) between **1a** and **2**, the averaged Fe—N bond lengths of *cis*-[Fe^III^(cyclam)Cl_2_]^+^ and *cis*-[Fe^III^(HMC)Cl_2_]^+^ are roughly 0.16 and 0.17 Å longer than those of *trans*-[Fe^III^(cyclam)Cl_2_]^+^ and *trans*-[Fe^III^(HMC)Cl_2_]^+^, respectively. The N—Fe—N angles of **1a**, *trans*-[Fe^III^(cyclam)Cl_2_]^+^, and *trans*-[Fe^III^(HMC)Cl_2_]^+^ round to 85° between the CH_2_CH_2_ linkage and 95° between the C*R*_2_CH_2_C*R*_2_ linkage. The N—Fe—N bond angles for the com­plexes with folded macrocycles vary from 79.56 (8) to 81.55 (5)° between the CH_2_CH_2_ linkage and from 83.15 (5) to 86.85 (15)° between the C*R*_2_CH_2_C*R*_2_ linkage. In short, each *cis* com­plex exhibits longer Fe—N bond lengths and N—Fe—N bond angles which deviate further from an ideal octa­hedral coordination geometry than in the respective *trans* com­plexes.

It is further possible to com­pare the structural parameters as the number of methyl groups is systematically varied within either the *cis* or *trans* series. The averaged Fe—N bond lengths of *cis*-[Fe^III^(HMC)Cl_2_]Cl (*ca* 2.21 Å) appears longer than those of **2** and *cis*-[Fe^III^(cyclam)Cl_2_]Cl (both *ca* 2.18 Å) (Clendening *et al.*, 2019 ▸; Guilard *et al.*, 1997 ▸), which is likely due to the steric bulk of the two methyl groups at the same position of the macrocycle (*e.g.* 5,5 and 12,12), which forces at least one methyl group to be axially oriented. For the *trans* structures, a continuous increase in the averaged Fe—N bond lengths may be observed from *trans*-[Fe^III^(cyclam)Cl_2_]FeCl_4_ (2.006 Å) to **1a** (2.024 Å) to *trans*-[Fe^III^(HMC)Cl_2_]FeCl_4_ (2.054 Å) (Clendening *et al.*, 2019 ▸; Guilard *et al.*, 1997 ▸). Although methyl groups are electron rich and would thus be expected to slightly increase the donor strength of the macrocycle towards the electron-poor Fe^III^ center, it appears that steric effects dominate, resulting in a progressively weaker Fe—N bond with increasing number of macrocylic substituents.

**Figure 7:**
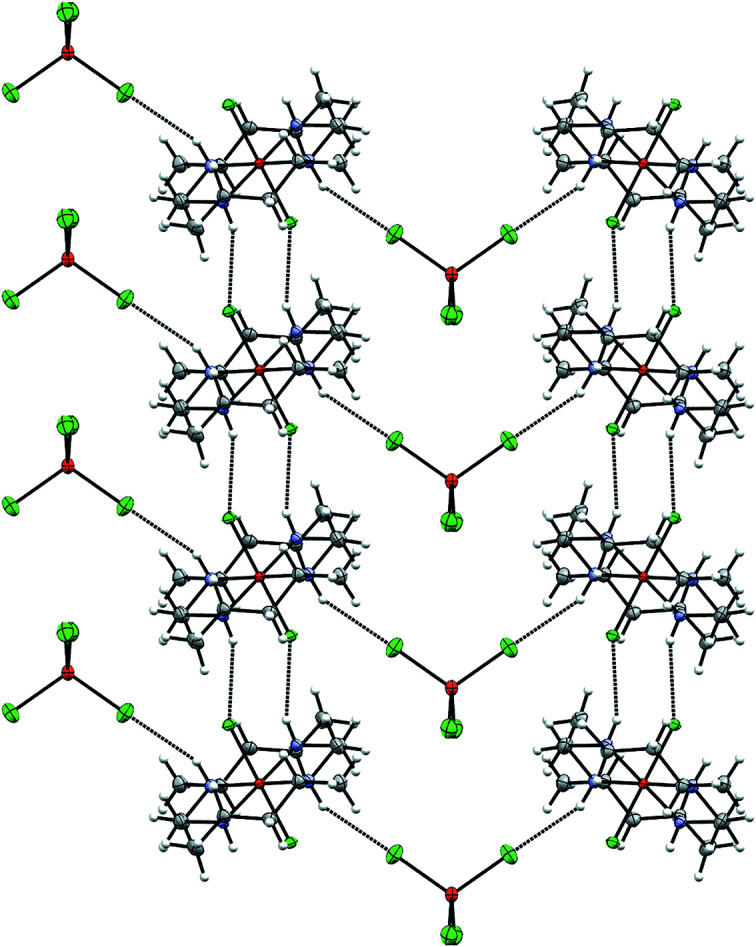
Illustration of the hydro­gen-bonding inter­actions in **1a**.

The packing in **1a** is strikingly similar to *trans*-[Fe^III^(HMC)Cl_2_]FeCl_4_ (Clendening *et al.*, 2019 ▸) and *trans*-[Fe^III^(cyclam)Cl_2_]FeCl_4_ (Guilard *et al.*, 1997 ▸), despite the differences in space group (*C*2/*c*, *P*2_1_/*c*, and *P*2_1_/*n*, respectively). The cat­ionic units in all cases pack in a series of columns (see Fig. 7 ▸), each linked to the preceding and following unit by two hydro­gen bonds between the amine groups and the chloride ligands (N1 and Cl1 in the case of **1a**). The remaining unique amine position(s) (N2 in **1a**) instead participates in hydro­gen-bonding inter­actions with the FeCl_4_^−^ units (*via* Cl3 in **1a**), which are also arranged in columns. This similarity suggests that the common tetra­chlorido­ferrate counter-anion may be favored due to this ability to participate in extensive hydro­gen-bonding networks with the ferric chloride com­plexes bearing planar macrocycles. The minor disordered counter-anion of **1a** has the same hydro­gen-bonding inter­actions as its major counterpart.

**Figure 8:**
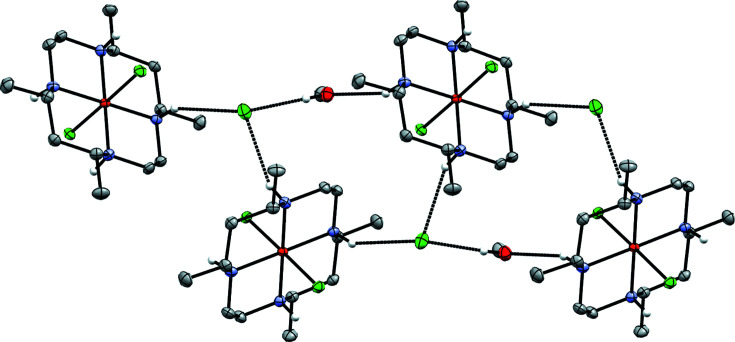
Illustration of the hydro­gen-bonding inter­actions in **1b**.

Lacking the FeCl_4_^−^ counter-anion, com­plex **1b** (*P*2_1_/*c*) packs differently, forming infinite chains parallel to [001] connected *via* hydro­gen bonds with one cation, one chloride counter-anion, and one methanol solvent mol­ecule as the repeat unit that are related to each other *via* one of the glide plane(s) (see Fig. 8 ▸). In between the glide planes and between neighboring chains, the mol­ecules are instead related by inversion. Inter­estingly, and in sharp contrast to the case of **1a** and its structural relatives, there are in **1b** no direct hydro­gen bonds between the cationic moieties (N1 is instead hydro­gen bonded to the methanol solvent mol­ecule and N3 and N4 to the chloride counter-anion), and both of the chloride ligands and one of the amine positions (N2) in **1b** do not participate in hydro­gen bonding at all.

This illustrates the difficulty of a single chloride counter-anion to effectively facilitate the hydro­gen bonding within the *trans* orientation of these com­plexes, apparently necessitating the solvent inclusion and further supporting the conjecture that the inclusion of FeCl_4_^−^ in the crystal structures is favored by its ability to facilitate ordered packing in the resulting solid. The packing differences may also hint at a supra­molecular origin for the elongated Fe—Cl and Fe—N bond lengths of the cationic moieties of **1b** *versus* **1a**, as the latter engages in more extensive hydro­gen bonding (six hydro­gen bonds per **1a** ^+^ and three per **1b** ^+^) which includes the chloride ligands, in contrast to **1b**.

**Figure 9:**
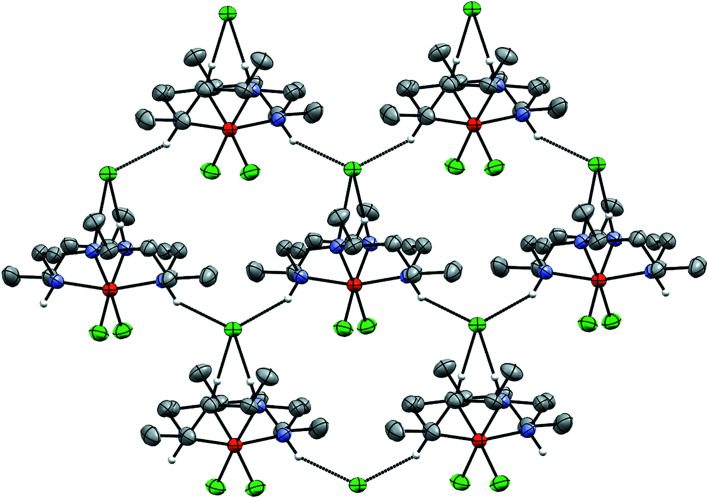
Illustration of the hydro­gen-bonding inter­actions in **2**.

**Figure 10:**
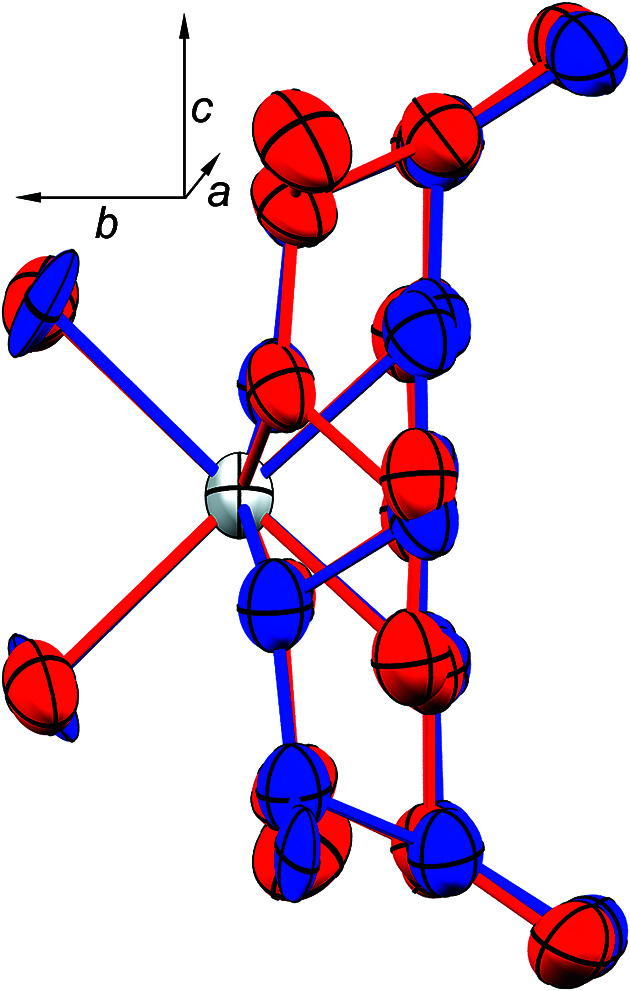
View of the major (red) and minor (blue) disordered moieties of **2** along the *a* axis, with the *b* axis oriented horizontally and the *c* axis vertically.

The similarity of the hydro­gen-bonding network among structural analogues extends to the cases of **2** (*Pbcn*), *cis*-[Fe^III^(HMC)Cl_2_]Cl (*Fdd*2), and *cis*-[Fe^III^(cyclam)Cl_2_]Cl (*P*2_1_/*c*) (Clendening *et al.*, 2019 ▸; Guilard *et al.*, 1997 ▸). As with **1b**, neither of the chloride ligands participates in hydro­gen bonding, nor do any of the cationic moieties hydro­gen bond with each other. However, in all three of these structures with a folded macrocycle, a close network of hydro­gen bonding is facilitated by the chloride counter-anion, which simultaneously participates in four such inter­actions with three separate cationic moieties (Fig. 9 ▸). Two hydro­gen bonds occur with the amines of a single moiety which face into the cavity of the folded macrocycle (N1 and its symmetry equivalent in **2**). The remaining hydro­gen bonds are formed from inter­actions with an outward-facing amine group of two separate moieties. Thus, in the case of the folded macrocycles, the large FeCl_4_^−^ anion is no longer necessary in order to stabilize an extended network of hydro­gen bonds. Inter­estingly, the minor disordered moiety of **2** maintains these strong hydro­gen-bonding networks, which perhaps facilitates the disorder about the *pseudo*-mirror plane in **2** (see Fig. 10 ▸).

## Conclusion

This article describes the structural properties of new ferric dichloride com­plexes of CTMC stereoisomers **A** (**1a**/**1b** – planar macrocycle) and **B** (**2** – folded macrocycle). DFT calculations are used to support the apparent preferences of **A** and **B** to form planar or folded coordination com­plexes, respectively. Moreover, com­parison to related structures based on cyclam and HMC illustrate a strong dependence of the inter­molecular hydro­gen-bonding inter­actions on the macrocyclic coordination geometry [*i.e.* planar (*trans*-di­chloro) or folded (*cis*-di­chloro)]. Given the known reactivity dependence of a com­plex on its overall geometry, com­plexes **1a**, **1b**, and **2** demonstrate how control can be exercised over the geometric coordination isomer by the stereochemistry of as few as four methyl groups on the periphery of the macrocycle. This contrasts with the ability of cyclam (no methyl groups) to assume either folded or planar conformations (Guilard *et al.*, 1997 ▸), and com­pliments the rigid structural preferences ascribed to the stereoisomers of HMC, which bears six methyl groups (Clendening *et al.*, 2019 ▸; House *et al.*, 1983 ▸; Tyler *et al.*, 2016 ▸). Work is underway to investigate derivatives of Fe(CTMC) with the goal of studying the charge-transfer excited states of such com­plexes.

## Supplementary Material

Crystal structure: contains datablock(s) 1a, 1b, 2, global. DOI: 10.1107/S205322962200849X/lf3128sup1.cif

Structure factors: contains datablock(s) 1a. DOI: 10.1107/S205322962200849X/lf31281asup2.hkl

Supporting information file. DOI: 10.1107/S205322962200849X/lf31281asup5.cdx

Structure factors: contains datablock(s) 1b. DOI: 10.1107/S205322962200849X/lf31281bsup3.hkl

Supporting information file. DOI: 10.1107/S205322962200849X/lf31281bsup6.cdx

Structure factors: contains datablock(s) 2. DOI: 10.1107/S205322962200849X/lf31282sup4.hkl

CCDC references: 2203222, 2203221, 2203220

## Figures and Tables

**Table 1:** Experimental details For all structures: *Z* = 4. Experiments were carried out at 150 K using a Bruker AXS D8 Quest diffractometer with a PhotonII charge-integrating pixel array detector (CPAD). Absorption was corrected for by multi-scan methods (*SADABS*; Krause *et al.*, 2015 ▸).

	**1a**	**1b**	**2**
Crystal data			
Chemical formula	[Fe(C_14_H_32_N_4_)Cl_2_][FeCl_4_]	[Fe(C_14_H_32_N_4_)Cl_2_]Cl·CH_4_O	[Fe(C_14_H_32_N_4_)Cl_2_]Cl
*M*_r_	580.83	450.68	418.63
Crystal system, space group	Monoclinic, *C*2/*c*	Monoclinic, *P*2_1_/*c*	Orthorhombic, *P* *b* *c* *n*
*a*, *b*, *c* (Å)	20.3512 (13), 6.4815 (4), 18.049 (1)	8.1632 (4), 20.8470 (12), 12.1387 (7)	9.2912 (12), 11.9579 (19), 17.267 (3)
α, β, γ (°)	90, 100.452 (3), 90	90, 95.024 (2), 90	90, 90, 90
*V* (Å^3^)	2341.3 (2)	2057.8 (2)	1918.4 (5)
Radiation type	Mo *K*α	Mo *K*α	Cu *K*α
μ (mm^−1^)	1.93	1.13	10.15
Crystal size (mm)	0.20 × 0.20 × 0.20	0.34 × 0.10 × 0.09	0.12 × 0.08 × 0.05

Data collection			
*T*_min_, *T*_max_	0.656, 0.747	0.679, 0.747	0.526, 0.754
No. of measured, independent and observed [*I* > 2σ(*I*)] reflections	71685, 4483, 3561	125711, 7859, 6505	14150, 2055, 1324
*R*_int_	0.074	0.049	0.082
(sin θ/λ)_max_ (Å^−1^)	0.771	0.770	0.638

Refinement			
*R*[*F* ^2^ > 2σ(*F* ^2^)], *wR*(*F* ^2^), *S*	0.027, 0.067, 1.02	0.024, 0.061, 1.04	0.066, 0.196, 1.08
No. of reflections	4483	7859	2055
No. of parameters	148	235	196
No. of restraints	15	0	273
H-atom treatment	H atoms treated by a mixture of independent and constrained refinement	H atoms treated by a mixture of independent and constrained refinement	H-atom parameters constrained
Δρ_max_, Δρ_min_ (e Å^−3^)	0.43, −0.48	0.70, −0.58	0.58, −0.95

Computer programs: *APEX3* (Bruker, 2019 ▸), *SAINT* (Bruker, 2019 ▸), *SHELXT2014* (Sheldrick, 2015*a* ▸), *SHELXT* (Sheldrick, 2015*a* ▸), *SHELXL2018* (Sheldrick, 2015*b* ▸), *SHELXLE* (Hübschle *et al.*, 2011 ▸), *Mercury* (Macrae *et al.*, 2020 ▸), and *publCIF* (Westrip, 2010 ▸).

**Table 2:** Selected geometric parameters (Å, °) for **1a**, **1b**, and **2**

Bond lengths	**1a**	**1b**	**2**	Bond angles	**1a**	**1b**	**2**
Fe1—Cl1	2.2710 (3)	2.3084 (3)	2.3018 (15)	N1—Fe1—N2	85.21 (4)	85.23 (3)	86.85 (15)
Fe1—Cl2	–	2.3047 (3)	–	N1—Fe1—N2^i,ii^	94.79 (4)	–	80.87 (16)
Fe1—N1	2.0276 (11)	2.0826 (9)	2.213 (4)	N1—Fe1—N4	–	94.13 (3)	–
Fe1—N2	2.0203 (11)	2.0787 (9)	2.154 (4)	N2—Fe1—N3	–	94.69 (3)	–
Fe1—N3	–	2.0654 (8)	–	N3—Fe1—N4	–	85.96 (3)	–
Fe1—N4	–	2.0761 (8)	–	Cl1—Fe1—Cl1^i,ii†^	180.0	179.012 (11)	91.97 (8)

Symmetry code for **2**: (i) −*x*, *y*, −*z* + 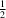 ; for **1a**: (ii) −*x* + 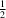 , −*y* + 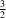 , −*z* + 1.

† Compound **1b**: Cl2—Fe1—Cl1.

## References

[bb1] Becke, A. D. (1993). *J. Chem. Phys.* **98**, 5648–5652.

[bb2] Bruker (2019). *APEX3* and *SAINT*. Bruker AXS Inc., Madison, Wisconsin, USA.

[bb3] Cao, Z., Forrest, W. P., Gao, Y., Fanwick, P. E. & Ren, T. (2012). *Organometallics*, **31**, 6199–6206.

[bb4] Clendening, R. A. & Ren, T. (2022). *Eur. J. Inorg. Chem.* **2022**, e202101021.

[bb5] Clendening, R. A., Zeller, M. & Ren, T. (2019). *Acta Cryst.* C**75**, 1509–1516. 10.1107/S2053229619013925 31686662

[bb6] Clendening, R. A., Zeller, M. & Ren, T. (2022). *Inorg. Chem.* **61**, 13442–13452. 10.1021/acs.inorgchem.2c01743 35916671

[bb7] Constable, E. C. (1999). In *Coordination Chemistry of Macrocyclic Compounds*. Oxford University Press.

[bb8] Dennington, R., Keith, T. A. & Millam, J. M. (2016). *GaussView*. Version 6. Semichem Inc., Shawnee Mission, KS, USA.

[bb9] Frisch, M. J., *et al.* (2016). *GAUSSIAN16*. Revision A.03. Gaussian Inc., Wallingford, CT, USA. https://gaussian.com/.

[bb10] Guilard, R., Siri, O., Tabard, A., Broeker, G., Richard, P., Nurco, D. J. & Smith, K. M. (1997). *J. Chem. Soc. Dalton Trans.* pp. 3459–3463.

[bb11] House, D. A., Hay, R. W. & Akbar Ali, M. (1983). *Inorg. Chim. Acta*, **72**, 239–245.

[bb12] Hübschle, C. B., Sheldrick, G. M. & Dittrich, B. (2011). *J. Appl. Cryst.* **44**, 1281–1284. 10.1107/S0021889811043202 PMC3246833 22477785

[bb13] Kolinski, R. A. & Korybut-Daszkiewicz, B. (1975). *Inorg. Chim. Acta*, **14**, 237–245.

[bb14] Kottrup, K. G. & Hetterscheid, D. G. H. (2016). *Chem. Commun.* **52**, 2643–2646. 10.1039/c5cc10092e 26751607

[bb15] Krause, L., Herbst-Irmer, R., Sheldrick, G. M. & Stalke, D. (2015). *J. Appl. Cryst.* **48**, 3–10. 10.1107/S1600576714022985 PMC4453166 26089746

[bb16] Macrae, C. F., Sovago, I., Cottrell, S. J., Galek, P. T. A., McCabe, P., Pidcock, E., Platings, M., Shields, G. P., Stevens, J. S., Towler, M. & Wood, P. A. (2020). *J. Appl. Cryst.* **53**, 226–235. 10.1107/S1600576719014092 PMC6998782 32047413

[bb17] Mash, B. L., Raghavan, A. & Ren, T. (2019). *Eur. J. Inorg. Chem.* **2019**, 2065–2070.

[bb18] Meyer, K., Bill, E., Mienert, B., Weyhermüller, T. & Wieghardt, K. (1999). *J. Am. Chem. Soc.* **121**, 4859–4876.

[bb19] Prakash, J., Rohde, G. T., Meier, K. K., Münck, E. & Que, L. J. (2015). *Inorg. Chem.* **54**, 11055–11057. 10.1021/acs.inorgchem.5b02011 26615667

[bb20] Rohde, J.-U., In, J.-H., Lim, M. H., Brennessel, W. W., Bukowski, M. R., Stubna, A., Münck, E., Nam, W. & Que, L. J. (2003). *Science*, **299**, 1037–1039. 10.1126/science.299.5609.1037 12586936

[bb21] Sheldrick, G. M. (2015*a*). *Acta Cryst.* A**71**, 3–8.

[bb22] Sheldrick, G. M. (2015*b*). *Acta Cryst.* C**71**, 3–8.

[bb23] Straub, S. & Vöhringer, P. (2021). *Angew. Chem. Int. Ed.* **60**, 2519–2525. 10.1002/anie.202012739 PMC7898313 33022879

[bb24] Tahirov, T. H., Lu, T.-H., Liu, G.-S., Chi, T.-Y. & Chung, C.-S. (1995*a*). *Acta Cryst.* C**51**, 1146–1148.

[bb25] Tahirov, T. H., Lu, T.-H., Liu, G.-S., Chi, T.-Y. & Chung, C.-S. (1995*b*). *Acta Cryst.* C**51**, 2018–2020.

[bb26] Tyler, S. F., Judkins, E. C., Song, Y., Cao, F., McMillin, D. R., Fanwick, P. E. & Ren, T. (2016). *Inorg. Chem.* **55**, 8736–8743. 10.1021/acs.inorgchem.6b01285 27529498

[bb27] Wang, J.-W., Liu, W.-J., Zhong, D.-C. & Lu, T.-B. (2019). *Coord. Chem. Rev.* **378**, 237–261.

[bb28] Weigend, F. & Ahlrichs, R. (2005). *Phys. Chem. Chem. Phys.* **7**, 3297–3305. 10.1039/b508541a 16240044

[bb29] Westrip, S. P. (2010). *J. Appl. Cryst.* **43**, 920–925.

